# Neuroprotective Effect of a Formula, Moschus Combined with Borneolum Synthcticum, from Traditional Chinese Medicine on Ischemia Stroke in Rats

**DOI:** 10.1155/2014/157938

**Published:** 2014-03-25

**Authors:** Xin-hua Xia, Qiang Li, Mei Liu

**Affiliations:** ^1^The First Hospital Affiliated to Guangzhou Medical University, Guangzhou 510120, China; ^2^The Heart Center, The First Hospital Affiliated to Lanzhou University, Lanzhou 710030, China; ^3^School of Chinese Material Medical of Guangzhou University of Chinese Medicine, 232 East Waihuan Road, Guangzhou Higher Education Mega Center, Guangzhou, Guangdong 510006, China

## Abstract

Moschus compatible with borneolum synthcticum is a well-known herb pair in Traditional Chinese Medicine and the present study aims to assess the neuroprotective effect of a formula composed of this herb pair on ischemia stroke in rats. The middle cerebral artery occlusion model of focal cerebral ischemia in rat was performed by using intraluminal suture method. The behavioral scores, infarct volume, and neuron ultrastructure of model and formula-treated rats were investigated after the 2 h of ischemia and 24 h of reperfusion. Meanwhile the expression levels of caspase-3, caspase-9, Bcl-2, and Bax were measured by western blot analysis. The formula treatment showed obvious neuroprotective effect according to significant decrease of the neurological scores (*P* < 0.01) and the infarct volumes (*P* < 0.05) when compared to the MCAO group. We also observed that this formula had antiapoptosis activity on neuron cell under electron microscope. Furthermore, our result supported the idea that pro- and postadministration of this formula had an antiapoptosis effect by decreasing remarkably the expression of caspase-3 and caspase-9 (*P* < 0.05) as well as increasing significantly the ratio of Bcl-2 to Bax (*P* < 0.01). All evidences demonstrated the neuroprotective effect of this formula on ischemia stroke due to decrease of brain infract volume and modulation of the expression of apoptosis-related proteins.

## 1. Introduction

Stroke is a cerebrovascular accident that is caused by either the ischemia or rupture hemorrhage of an artery to the brain and results in the interruption of blood flow to the brain [1]. Stroke is in the top three among all causes of death behind diseases of the heart and cancer [2]. Up to 87% of strokes are ischemia origin which is most attributable to the blockage of a artery by a blood clot [3]. Currently, the principle of clinical treatment to acute stroke is restoration of blood supply as soon as possible for resupplying oxygen to ischemic brain tissue [4]. At present, the only US Food and Drug Administration (FDA) approved treatment of tissue plasminogen activator (tPA) within 3 h of symptom onset is available to reestablish cerebral blood flow. However, this treatment has some limits of the short time window for tPA application, which is restricted to up to 3 hours after symptom onset [5]. As a result, a report from 2008 estimated that only 1.8 to 2.1% of all stroke patients had been treated with tPA in the United States [6]. On the other hand, some patients experienced disastrous outcomes in the form of fatal edema or intracranial hemorrhage following thrombolysis. Some researchers have reported that reperfusion caused by tPA or surgery after a long ischemic period can induce a larger infarct area than that associated with permanent vessel occlusion in some animal stroke models [7, 8]. Thus, while reperfusion may reduce infarct size and improve clinical outcome in some patients, in others it may exacerbate the brain injury and produce a so-called cerebral reperfusion injury [9, 10]. With the progress made in the understanding of the mechanisms in ischemia stroke and reperfusion injury, an increasing number of strategies have been developed for limiting or preventing further brain damage [11–13]. However, the translation of these promising strategies into effective therapies in humans has been disappointing. It is urgent to find therapeutic alternatives to treat ischemic stroke and reperfusion injury well.

Interestingly, prescriptions from Traditional Chinese Medicine (TCM) are made to treat all kinds of cerebrovascular diseases according to their experience and heritage from generation to generation over thousand years. A lot of studies have reported that some TCM preparations including Qing-Kai-Ling injection, An-Gong-Niu-Huang pill, Tong-Xin-Luo capsule, Nao-Shuan-Tong capsule, and Tong-Luo-Jiu-Nao capsule have been widely used in clinical practice to treat cerebrovascular diseases and showed effectiveness for the treatment of stroke by antioxidation, anti-inflammation, and neuroprotective function against ischemic reperfusion injury [14–19]. These preparations were composed of more than ten Chinese herbal medicines with a large number of complex components. Among them, the drug combination of moschus compatible with borneolum synthcticum was considered as the basic and primary unit of these preparations from the view of TCM. Moschus is a rare Chinese medicine, which is dry secretions and origins from mature male moschus deer including* Moschus berezovskii *Flerov,* Moschus sifanicus *Przewalski, and* Moschus moschiferus *Linnaeus. Moschus has been used as a common drug to awaken damaged brain in China with a long history. And borneolum synthcticum is the resin product of* Dryobalanops aromatica *Gaertn.f., which is also a regular refreshing and resuscitating drug commonly used for treatment of stroke, phlegm, coma, or related diseases. Both of them are generally used as a drug pair in TCM, and borneolum synthcticum mostly acts as an adjuvant or excipient in prescriptions, enhancing the efficiency of other drugs like moschus. We have extensively made a formula composed of moschus combined with borneolum synthcticum to treat the acute stages of cerebrovascular disease and have acquired excellent result on improving neurological function in clinical practise. Observing hundreds of cases of ischemic stroke in clinical practice, we have found that this formula used in early stage of ischemic injury could improve patients' awareness of barriers, shorten the treatment of ischemic stroke, and reduce a series of complications caused by the disturbance of consciousness. However, the specific neuroprotective pharmacological action and possible mechanism of this formula on ischemic stroke are still unclear and have not been reported yet. Therefore, we investigated the neuroprotective effects and possible molecular mechanism in order to offer a more effective therapeutic or alternative treatment for clinical practice of ischemic stroke. The model of ischemia stroke was performed in rats by middle cerebral ischemia reperfusion model (MCAO) method which was similar to the syndromes of clinical ischemia patients. The behavioral scores, brain infarct volume, and neuron ultrastructure of modeled rats and formula-treated rats were investigated to find out how the formula works to protect the brain function and repair the brain damage. And In order to evaluate the antiapoptosis activity of this formula, the expression levels of caspase-3, caspase-9, Bcl-2, and Bax also were measured by western blot analysis. With this information, we are hoping to offer some effective therapeutic alternatives to treat ischemia stroke, which will be an important theoretical significance and application value for further treatment of ischemic stroke.

## 2. Materials and Methods

### 2.1. Materials and Animals

Natural moschus (batch number: 20100322) and borneolum synthcticum (batch number: 20100413) were purchased from Chinese Herbal Medicine Co., Ltd., of Guangdong province, which were qualified by GC-FID according to Chinese Pharmacopeia 2010 edition. 6-0 nylon monofilaments for MCAO were bought from Beijing Shadong Biotech Inc. (Beijing, China); nitrocellulose membrane, 2,3,5-triphenyltetrazolium chloride (TTC), Tween 80, formaldehyde, chloral hydrate, and heparin were purchased from Sigma Company (St. Louis, MO, USA). Monoclonal antibodies of B-cell lymphoma 2 protein (Bcl-2), Bcl-2 associated X protein (Bax), caspase-3, caspase-9, and *β*-actin and horseradish peroxidase-conjugated IgG antibody were purchased from Nanjing Jianye Biotech Inc. (Nanjing, Jiangsu, China).

SD rats of SPF grade in either sex with the weight between 200 and 250 g (certificate number: SCXY-YUE-2003-0001) were purchased from Experimental Animal Center of Guangdong Province. All rats were housed in SPF grade animal room with temperature of 25 ± 2°C and relative humidity of 60% and were feed SPF grade rat food and sterile distilled water. All animal procedures involving animals and their care were conducted in accordance with the guidelines of Animal Use and Care of the National Institutes of Health (NIH Pub. number: 85-23, revised 1996) and were approved by the Animal Care and Use Committee of Guangzhou University of Chinese Medicine.

### 2.2. Middle Cerebral Ischemia Reperfusion Model and Experimental Groups

The middle cerebral artery occlusion (MCAO) model of focal cerebral ischemia in rat brain was performed by using intraluminal suture method [20]. Rats were fasted 12 h before experimental surgery with free access to water. Then all rats were anaesthetized with chloral hydrate (0.35 g·kg^−1^ body weight, i.p.) and were allowed to breathe spontaneously. Rats were placed in the supine position on the rat board with removal of hair in the center of neck. The right common carotid artery (CCA), external carotid artery (ECA), internal carotid artery (ICA), and the pterygopalatine artery were exposed and separated along the inner edge of sternocleidomastoid. Meanwhile vascular branch was being electrocauterized to prevent bleeding during separation. Then pterygopalatine artery and ECA were ligated at 1cm distal from the carotid bifurcation, and the distal end was burned off with coagulator. The CCA was occluded with aneurysm clips and a small incision was made in the ECA with syringe needle at about 0.5 cm away from ligation at the proximal end, and then pulling the proximal end of the external carotid artery in straight with the internal carotid artery was done. A nylon filament with silicone resin-coated tip (about 220 *μ*m in diameter and 4 mm in length) was inserted through the incision into the ICA about 18–20 mm. Reperfusion initiated by removing the filament after two hours of ischemia by blocking the artery, then the artery was ligated; meanwhile tissue and skin were sutured. The rats in sham group were conducted with the same procedure of rats in MCAO group but with no insertion of nylon filament. Body temperature of rat was kept at 36.5 to 37.0°C from the start of the surgery until the animal was awake from anesthesia. Neurological function was evaluated when the MCAO rats were awake, and those with scores less than 2 were excluded from the present study.

MCAO rats with neurological scores between 2 and 3 were randomly divided into three groups (8 rats for each group). The groups were denoted as sham group, vehicle-treated MCAO group (MCAO), and formula-treated group (MCAO+MB), respectively. Besides these three groups, other MCAO rats were divided into several subgroups (6 rats for each group) for the dosage and time window assessment. The drug injections were prepared by the First Hospital Affiliated to Guangzhou Medical University, which were composed of 1 mg·mL^−1^ of moschus and 3 mg·mL^−1^ of borneolum synthcticum. The dosages of drugs were calculated as equivalent dose as the usual dosage for clinical practice in hospital of TCM. The sham and MCAO group were administrated intraperitoneally normal saline of 10 mL·kg^−1^ body weight, the formula-treated group was administrated intraperitoneally drug injection with the content of 10 mL·kg^−1^ body weight, and the subgroups for dose assessment were administrated intraperitoneally drug injection with the content of 15 mL·kg^−1^, 10 mL·kg^−1^, and 5 mL·kg^−1^ body weight, respectively. The subgroups for time window assessment were administrated intraperitoneally drug injection with the content of 10 mL·kg^−1^ body weight. Each group was administrated intraperitoneally the correlated injection with a dose described above once at 30 min before ischemia and then repeated administration at 2 h, 12 h, and 24 h after ischemia.

### 2.3. Neurological Deficit Evaluation

Neurological deficits were measured after 2 h of ischemia and 24 h of reperfusion based on a five-point scale system reported in Xu's study [21]. Zero score was made for modeled rats with no obvious neurological deficits, one score was made for modeled rats with mild neurological deficits such as failure to extend contralateral forepaw on lifting of the animal by tail, two score was made for modeled rats with moderate neurological deficits including circling to the contralateral side but normal posture at rest, three score was made for modeled rats with severe neurological deficits such as falling to the contralateral side at rest, and four score was made for modeled rats with very severe neurological deficits of no spontaneous motor activity or even death.

### 2.4. Cerebral Infarct Volume Determination

The modeled rats were anaesthetized after 2 h of ischemia and 24 h of reperfusion. The brain of each rat was quickly removed from skull, rinsed with ice-cold saline immediately, and then put into the ice-cold saline for 15 min and after that the brain was cut into 2 mm slices. Slices were incubated with 2% TTC solution at 37.0°C for 30 min and then fixed by 10% formalin for 24 h. Finally, the stained brain slices were photographed in sequence with a camera after 24 hours of fixation. Areas of red and white staining were measured using an Image-Pro Plus6.0 (Media Cybernetics, Wyoming, USA). The percent of infarction size was calculated by the equation: %Infarct volume = Infarct volume/Total volume of slice × 100.

### 2.5. Ultrastructure Change of Neurons

The rat of each group was decapitated and the brain was quickly removed after 24 hours of reperfusion following MCAO injury. The right frontal cortex of brain tissue was cut into pieces of 1 mm^3^. All pieces were rapidly immerged and prefixed in cold 2.5% glutaraldehyde solution, then they were put and postfixed in 1% osmium acid solution. After fixation, the samples were dehydrated in 50%, 70%, 80%, 90%, and 100% ethanol, respectively. Finally, the dehydrated brain slices were embedded in phthalate propylene and then cut into 50 nm ultrathin sections. The sections were stained by uranyl acetate and lead citrate. The stained sections were observed by an electron microscope.

### 2.6. Western Blot Analysis

After 2 h of ischemia followed by 24 h of reperfusion, the whole right hemisphere tissue was lysed in lysis buffer for 15 minutes at 4°C and then centrifuged at 2060 ×g for 10 min. The supernatants were removed for western blot analysis. The same amount of total protein was isolated by sodium dodecyl sulfate polyacrylamide gel electrophoresis and then was transferred to a polyvinylidene fluoride membrane. This membrane was incubated with primary monoclonal antibodies of caspase-3, caspase-9, Bax, Bcl-2, and *β*-actin overnight at 4°C after blocking. The blots were washed with 5% dry milk in phosphate buffered saline/0.1% Tween 20 (PBST) and incubated with secondary antibody of horseradish peroxidase-conjugated IgG at room temperature for 1 hour. The protein bands were detected using an Amersham enhanced chemiluminescence detection system (GE Healthcare, Little Chalfont, UK). The protein expression of caspase-3, caspase-9, Bax, and Bcl-2 was analyzed with densitometry scans after normalization with the corresponding expression of *β*-actin.

### 2.7. Statistical Analysis

All data are expressed as means ± SEM. SPSS 19.0 (SPSS, Chicago, IL, USA) was used for statistical analysis. One-way analysis of variance was used followed by post hoc analysis for significance with the Student-Newman-Keuls multiple comparison test. *P* < 0.05 was considered statistically significant.

## 3. Results

### 3.1. Major Components of the Tested Formula Drug

**Figure 1:**
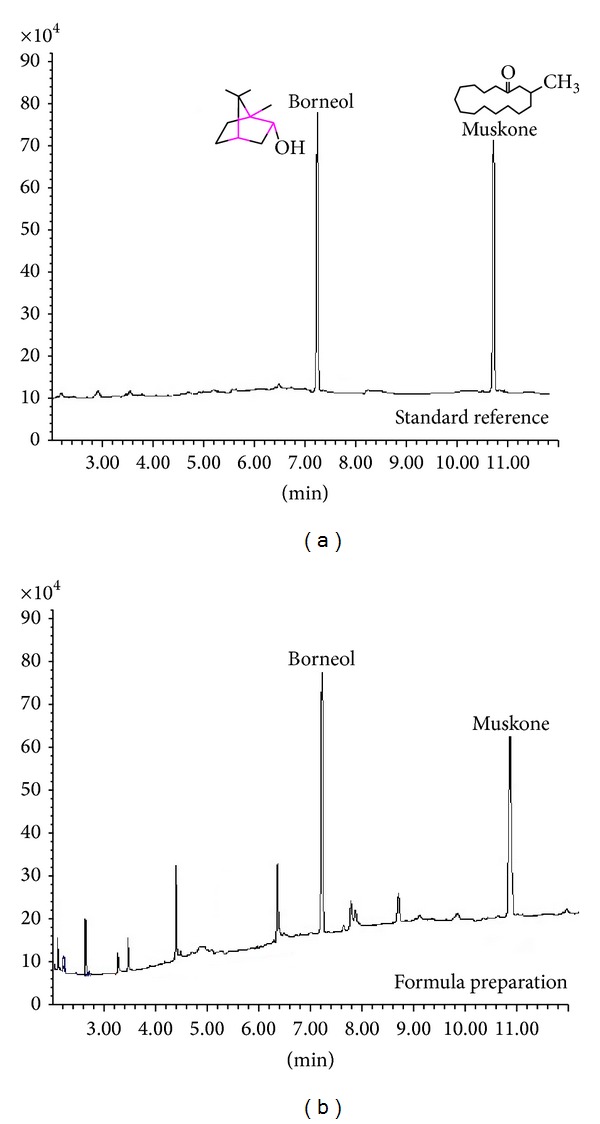
The major components of the formula were determined by GC-FID method as follows. Helium at a flow rate of 1.1 mL/min was used as the carrier gas. The initial oven temperature was 80°C, followed by a ramp to 180°C at 20°C/min, and then ramped to 280°C at 30°C/min (held at 280°C for 5 min). The injection port and detector temperatures were at 280°C, respectively. The hydrogen and air flow rates in FID 40 and 430 mL/min, respectively. (a) Was the GC-FID chromatogram of the standard references mixed with muscone and borneol. (b) Was the GC-FID chromatogram of the tested formula composed of moschus compatible with borneolum synthcticum.

The formula used in the present study was prepared by the First Hospital Affiliated to Guangzhou Medical University. The natural moschus and the geoauthentic borneolum synthcticum exported from Indonesia and named “Meipian” in Chinese Pinyin were used to prepare the formula injection, which is qualified by GC-FID following the related standards protocols of CHP 2010 and satisfied the standards in CHP 2010. The contents of muscone and borneol in the formula injection were determined by GC-FID following the related quantitative standards protocols established by our own lab (Figure 1). The muscone was the major component of moschus and its yield was of 5.04%, and the content of borneol was up to 89.6% in the injection formula. This formula contained 50.4 *μ*g of muscone and 2.69 mg of borneol per mL.

### 3.2. Treatments Decreased Neurological Behavioral Score and Reduced Brain Infarct Volume

**Figure 2:**
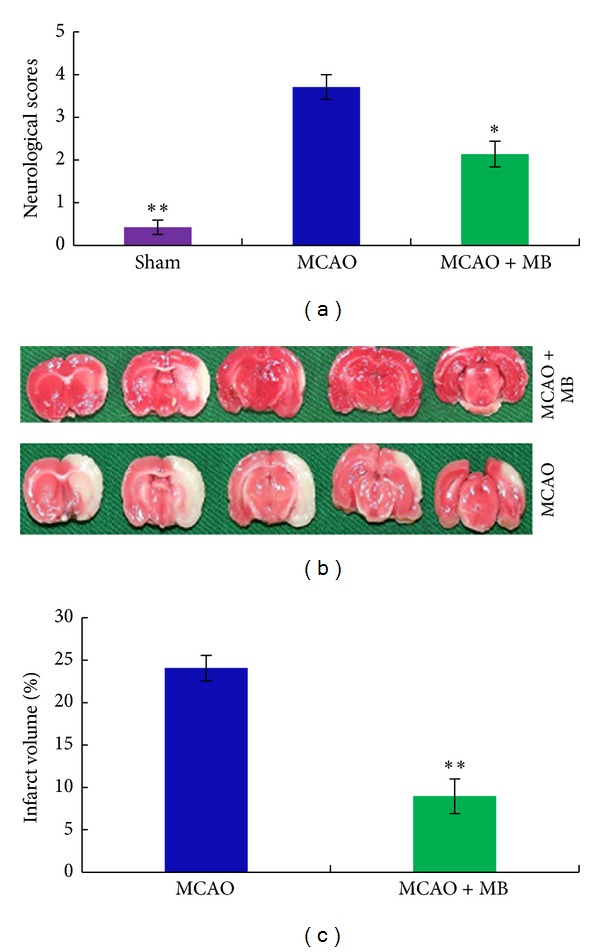
The comparison on the protective effects of treatments on the neurological deficits and the infarct volume after ischemia stroke. The rats were administrated intraperitoneally by the tested formula drug at 30 min before ischemia and at 2 h, 12 h, and 24 h after ischemia. After 2 h of ischemia and 24 h of reperfusion, the neurological scores were evaluated according to a graded scoring system described in the method in Section 2.3, and the infarct volume was calculated by the method described in Section 2.4. (a) Represented the different neurological deficits scores of sham group, MCAO group, and formula-treated group; (b) was the representative images of TTC-staining brains from MCAO group and the formula-treated group; the normal brain tissue displayed the color of rose red; meanwhile the infarct area displayed a color of pale white; (c) showed the different infarct volumes from rats of sham group, MCAO group, and formula-treated group. Bars represent means ± SEM of six rats; *significant difference at *P*≦0.05. **Remarkably significant difference at *P*≦0.01.

Before TTC staining, the neurological function score was calculated by behavior changes of animal with 5 levels. There were no obvious neurological deficits observed in rats from sham group. While in the MCAO group more severe neurological deficits were observed including circling movements, severe paw flections, and less spontaneous movements. All ischemia rats suffered from neurological impairment, and the scores of other rats were significantly higher than sham-operated rats. The neurological scores of formula-treated group declined significantly (*P* < 0.05) when compared with rats from MCAO group (Figure 2(a)). As shown in Figure 2(b), the image of the normal brain tissue displayed the color of rose red; meanwhile the image of the infarct area displayed a color of pale white after TTC staining. The area of pale white shrank evidently in ischemia rats of formula-treated group. In particular, the infract volume of rats from formula-treated group reduced significantly by 63% (*P* < 0.01) of the infract volume in rats from model group (Figure 2(c)).

### 3.3. Dose and Time Window Assessment on Infarct Volume

**Figure 3:**
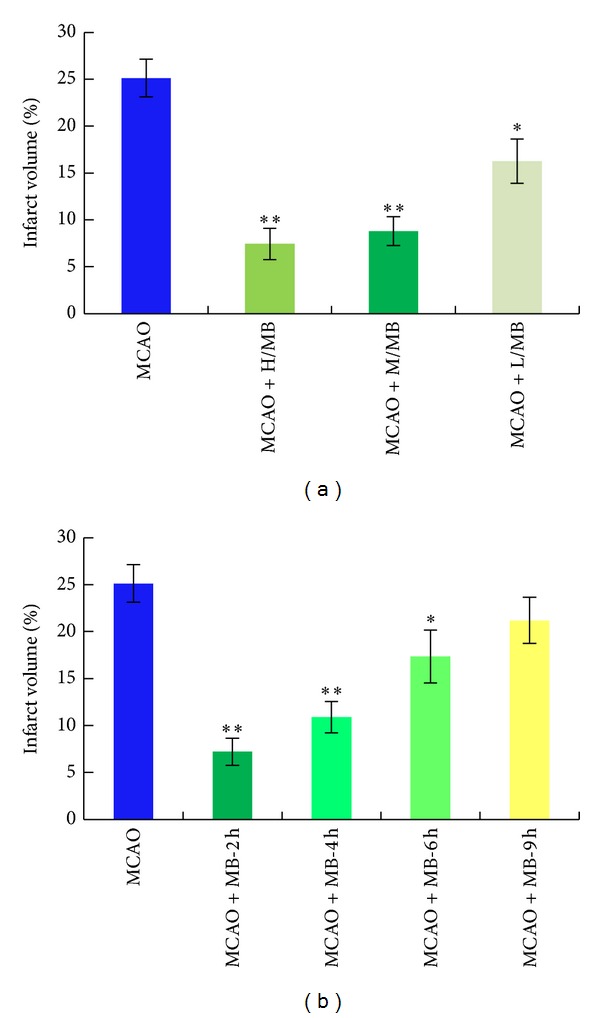
The results of dose-dependent and window time experiments: (a) the MCAO rats were administrated intraperitoneally with the prepared formula drug at high dose of 15 mL/kg body weight, moderate dose of 10 mL/kg, and low dose of 5 mL/kg. (b) The MCAO rats were administrated intraperitoneally 10 mL/kg at 2 h, 4 h, 6 h, and 9 h of ischemia. *Significant difference at *P*≦0.05 and **remarkably significant difference at *P*≦0.01.

In the dose-effect experiments, high dose, moderate dose, and low dose of the formula were set as the 1.5 times, 1 time, and 0.5 time of equivalent dose of the clinical dosage. Three subgroups were administrated intraperitoneally corresponding drugs with the content of 15 mL·kg^−1^ (MCAO+H/MB), 10 mL·kg^−1^ (MCAO+M/MB), and 5 mL·kg^−1^ (MCAO+L/MB). When compared with the infract size of rats from model group (Figure 3(a)), the infarction size of rats reduced significantly by 70% (*P* < 0.01), 64% (*P* < 0.01), and 35% (*P* < 0.05) in high, moderate, and low dose groups, respectively.

The time window was also evaluated in the present study. Injection of 10 mL·kg^−1^ formula drug was equivalent and effective dose of clinical dosage at reducing infarct volume and improving neurological function, so this dose was used in time window experiments. Four subgroups were administrated intraperitoneally with the formula drug injection at 2, 4, 6, and 9 h after ischemia, respectively. The formula treatment with different time significantly reduced cerebral infarct volume (*P* < 0.01 or *P* < 0.05, Figure 3(b)). The maximum reduction of infarct volume was statistically significantly decreased by 71% in the 2 h group compared to that in model group (*P* < 0.01). The infarct volumes gradually decreased significantly by 56% (*P* < 0.01) and 31% (*P* < 0.05) in the 4 h and 6 h treatment groups, respectively. However, the cerebral infarct volume of rats in 9 h group was 15% lower than that in model group with no significance.

### 3.4. Treatments Repaired Ultrastructure Change of Neurons

**Figure 4:**
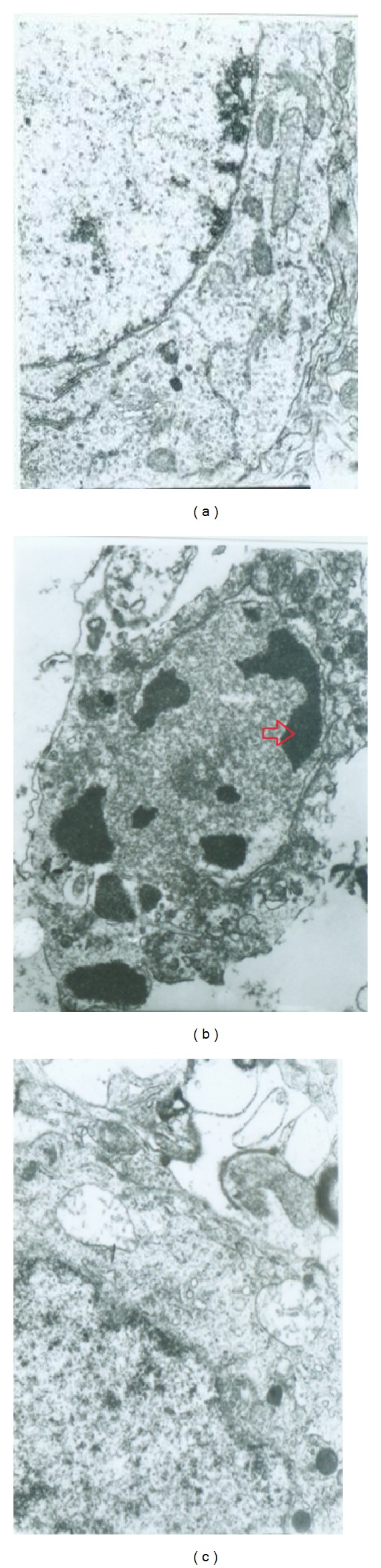
The representative images of neurons in MCAO rats' brains from different groups under electron microscope (×12 k). (a) Was a representative image of neuron cell from sham group, (b) was an representative image of neuron cell from MCAO group, and (c) was a representative image of neuron cell from formula-treat group.

The ultrastructural change of neurons was enlarged and observed by the electron microscope. As shown in Figure 4(a), the double membranes of the nerve cells in brain tissue from the sham group were clear to distinguish, and the nuclear of these cells was large and clear without nuclear chromatin condensation. There was much endoplasmic reticulum in cytoplasm and normal mitochondria without lesions around the nucleus.  Figure 4(b) showed that the nerve cells of brain tissue damaged by ischemia from the MCAO group shrank obviously, both of the cell membrane and the nuclear membrane were blurred, and there were nuclear chromatin agglomerated, mitochondria swelled, rough endoplasmic reticulum expanded, and gap around nerve cells increased obviously observed under the electron microscope. However, the nerve cells of brain tissue with ischemic injury treated by the formula drug had integral double nuclear membrane. There were no nuclear chromatin condensation and no obvious expansion of cytoplasm endoplasmic reticulum observed under electron microscope, and little mitochondria swelled (Figure 4(c)).

### 3.5. Western Blot Analyses

**Figure 5:**
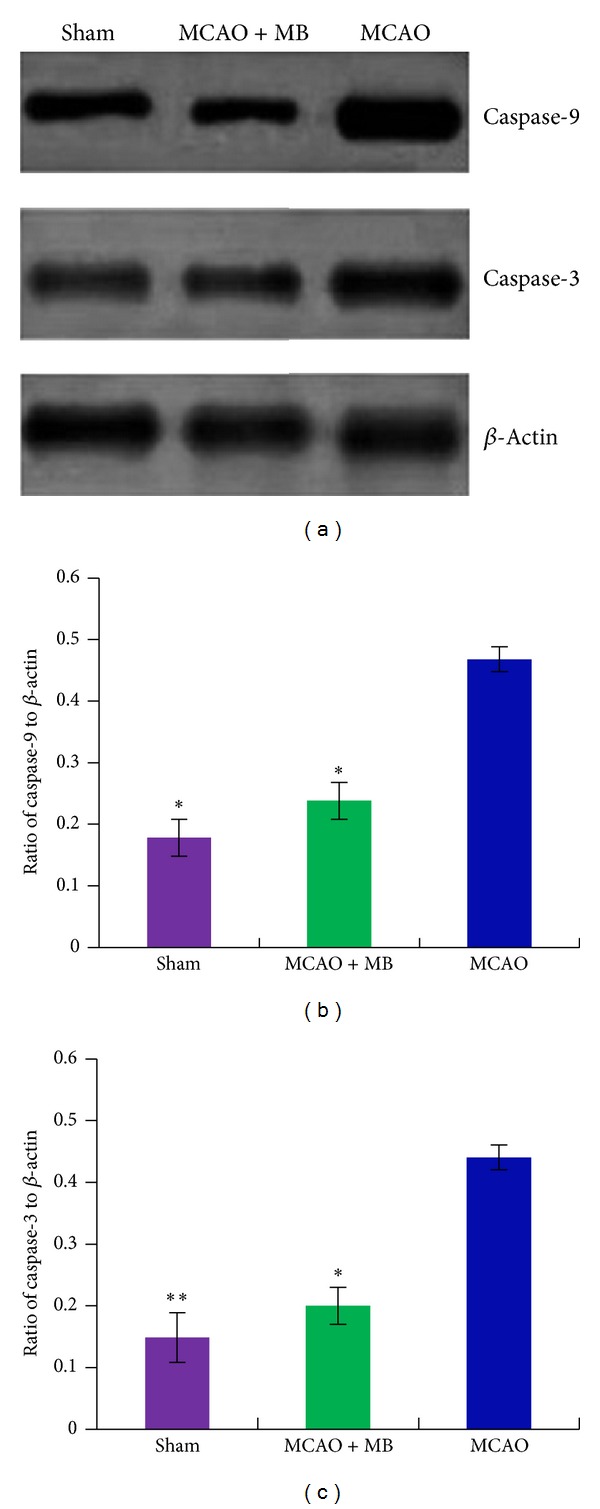
The comparison on the expression levels of caspase-3 and caspase-9 in the brain tissue after ischemia. (a) Was the representative graphs of caspase-3 and caspase-9, and *β*-actin was used as loading control. (b) Showed quantitative comparison on ratio of caspase-3 to *β*-actin among different groups; (c) showed quantitative comparison on ratio of caspase-9 to *β*-actin among different groups. Bars represent means ± SEM of eight rats. *Significant difference at *P*≦0.05. **Remarkably significant difference at *P*≦0.01.

Next, the molecular mechanism of neuroprotective activity induced by the formula was investigated by western blot analysis in MCAO rats. More and more evidences have revealed that different kinds of cell death including necrosis, necroptosis, and apoptosis occurred after cerebral ischemia injury [22, 23]. We also observed the different morphologic change in neuron cells after ischemia and treatment. Therefore, some markers protein of cell apoptosis like caspase-3 and caspase-9 were measured by western blot analysis. After 2 h of ischemia and 24 of reperfusion, the brain tissue of rats from all groups was collected and prepared for western blot analysis. As shown in Figure 5(a), there was significant increase of caspase-3 and caspase-9 expression levels observed in rats brain tissue from MCAO group, which confirmed that the neuronal cell apoptosis happened during the period of ischemia stroke. The treatment of the formula drug reduced remarkably the expression of caspase-3 (*P* < 0.05, Figure 5(b)) and caspase-9 (*P* < 0.05, Figure 5(c)) indicating that this formula drug has an antiapoptosis activity.

**Figure 6:**
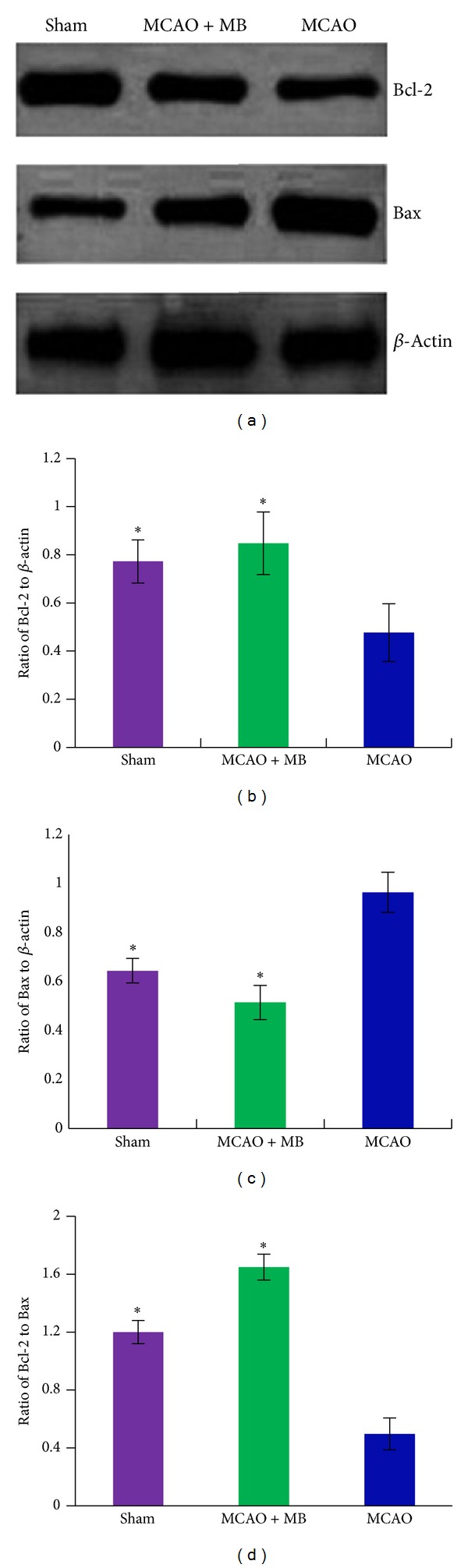
The comparison on the expression levels of Bcl-2 and Bax in the brain tissue after ischemia. (a) Was the representative graphs of Bcl-2 and Bax, and *β*-actin was used as loading control. (b) Showed quantitative comparison on ratio of Bcl-2 to *β*-actin among different groups, (c) showed quantitative comparison on ratio of Bax to *β*-actin among different groups, and (d) was quantitative comparison on ratio of Bcl-2 to Bax. Bars represent means ± SEM of eight rats. *Significant difference at *P*≦0.05. **Remarkably significant difference at *P*≦0.01.

Furthermore, the expression levels of some apoptosis-related proteins, such as Bcl-2 and Bax which are considered as the most important determining factors for the fate of cells in response to apoptotic stimulation. The ischemic brain samples from all groups were used to determine the protein levels of Bcl-2 and Bax by western blot analysis (Figure 6(a)). The formula treatment significantly increased expression level of Bcl-2 (*P* < 0.05, Figure 6(b)) and decreased expression level of Bax (*P* < 0.05, Figure 6(c)) after ischemia. The ratio of Bcl-2/Bax was drastically increased in formula-treated rats (*P* < 0.05, Figure 6(d)).

## 4. Discussion

Although dramatic progress has been made to alleviate the impact of stroke on public health and reduce stroke incidence and mortality, unfortunately, most therapeutic approaches developed in the laboratory have failed in large clinical trials. Therefore, translational stroke research requires a revaluation of traditional approaches and the development of a new conceptual framework to guide therapy. Increasing numbers of stroke patients have sought TCM therapy to improve physical functions in recent years that is worth our attention [24]. There are more and more evidence-based studies that have shown TCM's beneficial effects in stroke patients [25–27]. In this context, there is much to be gained by learning how the traditional therapies work to treat ischemia stroke and understanding how these protective measures provide useful lessons on how to best counteract ischemic brain injury. This paper aimed to provide an effective formula with possible mechanisms and highlighted the potential application for the future of stroke therapy. In China, TCM have been used to treat stroke with a long history over 2000 years. In recent decades, patent medicines of TCM (TCPM) and acupuncture were widely and regularly used in stroke patients in either western medicine hospitals or traditional Chinese medicine hospitals [28, 29]. Currently, there are more than 100 TCPM used for stroke and approved by the Chinese State Food and Drug Administration [30].

As a highly valued ingredient of Chinese medicinal remedies, moschus is a detoxification agent for treating inflammation, relieving swelling, and killing pain, which has been used widely in the important therapies of stroke, coma, and convulsion in clinical practice of the traditional hospitals. The administration of moschus extract has been recommended and listed in the CHP and the Japanese pharmacopoeia for various indications requiring cardiovascular stimulation, anti-inflammatory medication, or androgenic hormone therapy [31]. Borneolum synthcticum is also a common drug used often to treat coma, loose heat, brighten eyes, relieve swelling, and kill pain in TCM. Some reports have shown that borneolum synthcticum was used as one of the most effective ingredients of the traditional medicines which treat and prevent cardiovascular diseases like coronary artery disease, heart stroke, and heart infarction for its properties of antiapoptosis, anti-inflammation, and abirritation [32–34]. In addition, the borneolum synthcticum is also considered as a great promoter or enhancer to guide other drug to disease position in the theory and practice of TCM. Therefore, the polyherbal classical formulations are generally borneolum synthcticum combined with other ingredients to enhance the therapeutical effect of the treatments which especially often used in the treatments for cardiovascular and cerebrovascular diseases in the clinical practice of TCM. Many researchsd have also approved that the borneolum synthcticum has a beneficial effect on increasing the bioavailability, tissue distribution, and blood concentration of other drugs and makes other drugs transport through BBB easier [35]. In the present study, the formula tested in animal experiments originates from An-Gong-Niu-Huang pill which contains more than ten herbal agents and has very clear therapeutical effect on stroke in clinical practice of TCM. The moschus compatible with borneolum synthcticum at ratio of 1 : 1 in this patent drug was consider the primary drug combination and called “monarch drug” in this drug. As the basis of this ratio, we also investigated the neuroprotective effect of different ratio on ischemia stroke and found that the ratio of 1 : 3 was the most effective to reduce infract size in animal experiments and to improve the syndromes of patients who suffered from stroke in clinical practice. Therefore, we made a formula composed of moschus compatible with borneolum synthcticum at a ratio of 1 : 3, and the present study also demonstrated for the first time that this formula had neuroprotective effect on protecting brain function and repairing the brain damage from ischemia stroke through significantly reducing the infract volume and improving neurological deficits.

This formula has been prepared for injection and named “She-bing injection” by our own lab. The method for quality control of this injection was established in order to guarantee its therapeutical effect and safety. The muscone and borneol are the major components of this formula and were chosen as the chemical markers to control the quality of this injection. The GC-FID was performed to simultaneously determine the quantitative contents of muscone and borneol. The standard curves for the tested components were linear over the studied concentration ranges with correlation coefficient ≥0.9998. The limits of qualification for muscone and borneol were 0.021 and 0.014 *μ*g·mL^−1^, respectively. The RSDs of analytical accuracy and precision for two analytes are all lower than 3.0%. The recoveries of two analytes were from 90.21% to 98.6%. These results indicated that the developed method was accurate enough for the quality control of this injection.

Behavioral examination of neurological function score and determination of brain infarct volume in rats after ischemia and/or reperfusion are commonly used as the indicators to evaluate the ischemia injury and the severity of brain injury. Considering the dose-dependent and time window studies, it is showed that the neurological function impairment and infarct volume reached the peak after 2 h of focal cerebral ischemia followed by 24 h of reperfusion. Therefore, the same time was selected as time window for this study. The brain regions with a blockage of arterial blood involved in regulating movement, and therefore the animals with ischemia can show movement disorder after being awake. The right artery in ischemia reperfusion caused the same side motion control injuries, because the cortex had a function of cross-control, which leads to left limbs muscle weakness, and the rats rotated to the left when moving and displayed typical symptoms of rear-end and leaning to the left. Neurological function scores of the drug-treated rats dropped significantly lower than the MCAO group. It was indicated that the drugs could improve neurological dysfunction in focal cerebral ischemia-reperfusion rats.

Furthermore, we investigated the dose and time window of this formula based on the effect of reducing infract volume in the MCAO modeled rats. And the results indicated that this formula had a broad time window with a significant therapeutical effect in 6 hours after ischemia in MCAO rats, which suggested that this formula could have more effect on those patients who were excluded from the tPA treatment because of the strict limit of symptom onset time.

This formula is an important agent affecting many central nervous system functions in clinical practice. Its neuroprotective effects have been documented in present study by lesion model of experimentally induced ischemia. The evidences described above now suggested that this drug formula can significantly decrease lesion volume and improve functional recovery after stroke. Although the concrete mechanisms underlying the neuroprotection against cerebral ischemia by this formula are not completely known, some researchers have reported that neuronal cell death in the hippocampus might occur via apoptosis, a consequence of ischemia [36]. In our previous study, we also found that this formula had a rescue effect on cerebral ischemia by decreasing neuronal apoptosis with observation under electron microscope. We further explored the protective mechanism of this formula drug in cell apoptosis and assessed its influence on the expression level of apoptosis-related proteins like caspase-3, caspase-9, Bcl-2, and Bax in MCAO model rats. Our result supported that pro- and postadministration of this formula drug had an antiapoptosis activity by decreasing remarkably the expression of caspase-3 and caspase-9 as well as increasing the ratio of Bcl-2 to Bax. Protective effects of this formula with a broader time window offered a promise to treat stroke patients as a neuroprotective agent in clinical trials. Therefore, with these experimental data, we are hoping to find some more effective drugs to treat ischemia stroke better, which will be an important theoretical significance and application value for further treatment of stroke.

## 5. Conclusion

In the present study, the model of focal cerebral ischemia reperfusion was performed in rats by MCAO method. The behavioral scores, brain infarct area size, and neuron ultrastructure of modeled rats and formula-treated rats with focal cerebral ischemia or reperfusion injury were investigated to find out how the drug formula worked to protect and improve the brain function. And the results showed that this formula could significantly ameliorate neurobehavioral disturbances, shrink relative infarct size, and rescue neural dysfunction effectively. Furthermore, the formula could prevent neuron cells from apoptosis caused by cerebral ischemia or reperfusion to relieve brain damage. The possible mechanism of this neuroprotective effect on ischemia stroke could be the antiapoptosis activity by decreasing the expression of caspase-3 and caspase-9 as well as increasing the ratio of Bcl-2 to Bax.
